# Insight into the Candidate Genes and Enriched Pathways Associated with Height, Length, Length to Height Ratio and Body-Weight of Korean Indigenous Breed, Jindo Dog Using Gene Set Enrichment-Based GWAS Analysis

**DOI:** 10.3390/ani11113136

**Published:** 2021-11-02

**Authors:** Sunirmal Sheet, Jong-Seok Kim, Min-Jeong Ko, Na-Yeon Kim, Young-Jo Lim, Mi-Rim Park, Seung-Jin Lee, Jeong-Min Kim, Seok-Il Oh, Bong-Hwan Choi

**Affiliations:** 1 Animal Genome and Bioinformatics, National Institute of Animal Science, RDA, Wanju 55365, Korea; sunirmal.micro@gmail.com (S.S.); cruel022233@gmail.com (M.-J.K.); ks901223@gmail.com (N.-Y.K.); topair707@korea.kr (Y.-J.L.); cocci@korea.kr (M.-R.P.); 2 Korean Jindo and Domestic Animals Center, Jindo 58915, Korea; dvm10000@korea.kr (J.-S.K.); jindo1315@jindomail.net (S.-J.L.); kjm434364@naver.com (J.-M.K.)

**Keywords:** indigenous breed, Jindo, height, body-weight, length, GWAS, gene-set enrichment analysis, heritability, hormone synthesis pathways

## Abstract

**Simple Summary:**

The height, length and body-weight are the economic traits of Jindo dogs and it is necessary to study their underlying genetic basis. Thus, a post-genome-wide association enrichment study on four body-size traits of Jindo dog including height, length, length to height ratio and body-weight was carried out here. The results revealed several potential single nucleotide polymorphisms (SNPs) and genes which were significantly associated with the traits of interest. Furthermore, we have found several pathways to be significantly associated with the traits of interest and these pathways are generally involved in the growth and development process. The findings of this study may help to use the identified SNPs and genes as possible biomarkers for Jindo breeding.

**Abstract:**

As a companion and hunting dog, height, length, length to height ratio (LHR) and body-weight are the vital economic traits for Jindo dog. Human selection and targeted breeding have produced an extraordinary diversity in these traits. Therefore, the identification of causative markers, genes and pathways that help us to understand the genetic basis of this variability is essential for their selection purposes. Here, we performed a genome-wide association study (GWAS) combined with enrichment analysis on 757 dogs using 118,879 SNPs. The genomic heritability (h^2^) was 0.33 for height and 0.28 for weight trait in Jindo. At *p*-value < 5 × 10^−5^, ten, six, thirteen and eleven SNPs on different chromosomes were significantly associated with height, length, LHR and body-weight traits, respectively. Based on our results, *HHIP*, *LCORL* and *NCAPG* for height, *IGFI* and *FGFR3* for length, *DLK1* and *EFEMP1* for LHR and *PTPN2*, *IGFI* and *RASAL2* for weight can be the potential candidate genes because of the significant SNPs located in their intronic or upstream regions. The gene-set enrichment analysis highlighted here nine and seven overlapping significant (*p* < 0.05) gene ontology (GO) terms and pathways among traits. Interestingly, the highlighted pathways were related to hormone synthesis, secretion and signalling were generally involved in the metabolism, growth and development process. Our data provide an insight into the significant genes and pathways if verified further, which will have a significant effect on the breeding of the Jindo dog’s population.

## 1. Introduction

The Jindo dog is a magnificent Korean indigenous breed that originated from the Jindo county of South Korea. It has been highly regarded for its hostile disposition and watchfulness characteristics. It is well known as a loyal companion, independent hunter and discerning guardian. In 1998, the Jindo dog breed got recognition from United Kennel Club and Federation [1]. This breed has dark brown bright coloured eyes, small erected triangular ears and rolled up or sickle-shaped tails with acute hearing and sense of smell [2]. Jindo dogs are a double-coated spitz-type dog which range from 45 to 53 cm in height, and the body-weight range is 16 to 22 kg [2]. The majority of Jindo owners keep them for farmhouse income, as a watchdog and hunting. These days, the dogs are intensively farmed and kept as affectionate companion dog. The Jindo dog ranked 8th in the list of canine breeds kept as pets, falling behind other foreign breeds, and was found in only 4% of households that had a domestic dog [3].

Throughout the journey to domestication, the dog has become a part of the different functional and emotional needs of humans [4]. Domestic dogs nowadays are descended from random breeding or purpose-bred populations in which the selection of their parents is under humans control [5,6]. The intense artificial selection has resulted in a diverse range of dogs that serve as herders, hunters, protectors, trackers, support dogs, athletes, and most popularly, as companions [4]. Specifically, the purpose-bred dogs were produced for performing different highly specialised tasks [6,7,8]. For example, certain dogs were used to carry or pull bulky loads and consequently were selected for larger overall size and bones [6,9]. Some dogs were designated for speed to hunt or herd game and thus have longer legs and leaner builds [6,9,10,11]. Besides these functional purposes, various breeds were produced exclusively as companion animals [6,9]. In such circumstances, morphology was subjected to fewer restrictions, allowing for the selection of more extremes as required. Body sizes in domestic dogs can vary up to 50-times in terms of body weight (in Kg), from toy breeds to giant breeds [2].

One of the most visual examples of artificial selection for domestic dogs is body size variations including changes in height, length and body weight across [2,12,13,14]. The height, weight and other characteristics of the body’s size are the classic polygenic and highly heritable morphologic traits, influenced by several variants across different genetic loci [14,15,16,17]. Therefore, studies on a better understanding of the genes and variants that affect morphological variation in domestic dogs can be found from the late 19th century by Charles Stockard in [18] to today by modern canine geneticists.

A genome-wide association study (GWAS) is a powerful approach used in genetic research for identifying causal genes and loci associated with complex diseases and quantitative traits [19]. In recent years, the decreasing cost of high-throughput genotyping technology integrated with the ongoing gene set enrichment analysis (GSEA) has led to a boom in the number of GWAS. Genome-wide association studies in companion animal, including the dog, have provided significant insights into susceptible genes and genomic loci associated with numerous canine disease traits and many Mendelian disorder-related traits [20,21,22,23]. In addition to complex disease traits, GWAS have also been used to discover genes affecting different polygenic morphological traits in dogs as well as many other mammals and, of course, humans [15,23,24,25,26,27]. For example, a total of 13 loci have been discovered in dogs that have a significant impact on their weight and/or height [14,17,24,28,29,30,31,32,33,34,35]. Among the 13 loci, six loci have been reported for accounting for more than 80% of the diversity in body size trait in purebred dogs [24,25]. On the other hand, GWAS in humans based on tens of thousands to hundreds of thousands of samples so far revealed ∼700 loci for human height explaining only 20% of the variation [15,23].

In this study, we performed a GWAS analysis to investigate the candidate genes and biological pathways related to quantitative traits (height, length, length to height ratio (LHR) and body-weight) of 757 Korean Jindo dogs using SNP genotyping data from Illumina CanineHD BeadChip array and supplemented it with gene-set and pathway-based functional analysis. The significantly associated SNPs, candidate genes, gene ontology (GO) and pathways related to height, length, LHR and body weight were detected and thus, giving references for use during the marker-assisted selection of these traits in Jindo dog.

## 2. Materials and Methods

### 2.1. Animals and Phenotype Assignment

Institutional Animal Care and Use Committee (IACUC) approval at the National Institute of Animal Science (NIAS, RDA), was obtained for this study. The protocol consent was obtained for the ‘Development of early diagnosis technology for degenerative muscular skeleton system in special-purpose dog’ project. A total of 757 Jindo dogs were arbitrarily selected from the different farms and breeding centres within Jindo county of South Korea in the present study without any pedigree information. All dogs were selected without obesity-related diseases which could affect body weight and kept under fasting for 12 h from their last meal to get the dog’s true weight in the morning. The blood sample was collected at the same time of weight measuring. A dog’s height measurement was taken from the withers’ highest point down to the paws. The length measurement was taken from starting at the centre of the chest along the side of the body to the tail. The height and length were measured in centimetre (cm) and weight was measured in kilogram (kg). The following formula was used to get the LHR-

(1) $$\mathrm{LHR}=\left(\frac{\mathrm{Length}}{\mathrm{Height}}\right)\times 100$$

The other relevant information (e.g., identification number, intake date, age, sex and obesity-related) was retrieved from shelter records.

### 2.2. Genomic DNA Extraction, SNP Genotyping and Quality Control

We extracted genomic DNA from collected blood samples of 757 dogs, using the DNeasy Blood and Tissue Kit (Qiagen, Valencia, CA, USA). The samples were genotyped on a 170K Illumina CanineHD BeadChip (Illumina, San Diego, CA, USA) array, which contains 173,662 SNPs. The filtering criteria were set as follows: minor allele frequency < 5%, low genotyping call rate < 90%, missing genotype calls > 10%, Hardy–Weinberg equilibrium at *p* < 1 × 10^−6^ for quality control (QC) with PLINK v.1.9 software [19]. The final genotyping call rate was 98.75%. After QC filtering, 118,879 SNPs and 757 animals remained for further association analysis.

### 2.3. Genome-Wide Association Analysis

The association between traits and SNPs were tested using a mixed linear model approach implemented in GCTA v.191.4 beta3. Significant factors such as age (1–12), sex (492 females and 265 males) and birth year (2006–2019) were fitted in the GWAS statistical model as a fixed effect for all the traits. In GWAS, we generated a total of 20 principal components (PCs); the eigenvalues of all the PCs were fit as co-variance to account for population stratification. The GWAS statistical model used was as follows:

*y* = Z*μ*+ *Xb* + *g* + *e* (2)

where, *y* is a phenotypic trait, *μ* is additive genetic effect for each marker, *b* is an additive effect (fixed effect) including age, sex and birth year; *X* and Z are incidence matrices for the vectors of parameters *b* and *μ*, respectively; *g* is the accumulated effect of all the SNPs captured by the GRM (genetic relationship matrix, calculated using all the SNPs) and e is a vector of residual effect [19]. The significance threshold for GWAS was distinct using the Bonferroni correction method. A Bonferroni-corrected threshold was used to correct for multiple testing. The 5% genome-wide significance threshold was set at *p* < 4 × 10^−^^7^ (~0.05/118,879). However, this Bonferroni-corrected threshold was too stringent in this study and therefore, it might yield many false-negative results. Hence, the suggestive significance threshold value was set at *p*-value of <5 × 10^−^^5^ [36]. Further, Manhattan and quantile-quantile (Q-Q) plots were generated using the CMplot package in R [19]. The estimate of genotypic variance (V(G) and phenotypic variance (Vp) was performed using restricted maximum likelihood analysis (REML) implemented in GCTA v.191.4. beta3, while heritability (h^2^) was calculated using h^2^ = V(G)/Vp [37].

### 2.4. Gene Mapping, GO and Pathway Analysis

We performed a gene-set enrichment and pathway analysis for each trait following the methods described by Dadousis et al. [27]. We used a nominal *p*-value of <0.01 to filter SNPs from the GWAS for gene-set and pathway analysis. If the annotation of genes using only significant SNPs is carried out, it may not encounter the SNPs with less significant level. As a result, it will miss key putative genes and allied pathways [36]. Moreover, it has been shown that merging less significant but associated SNPs can provide information on how these markers might be collectively related to our phenotypes of interest [20]. The SNPs were annotated to genes within 5-kb flanking region using SnpEff version 4.3 software [19] and the genes were used in the enrichment analysis. The latest version of the *Canis lupus familiaris* (dog) genome assembly CanFam3.1 was used as the reference genome.

Genes name assigned to SNPs was filtered using SNP ID’s from variant call format (VCF) file that was used for mapping. For functional gene ontology (GO) and Kyoto Encyclopedia of Genes and Genomes (KEGG) pathway analysis, the annotated genes were then uploaded to the database for annotation, visualisation and integrated discovery (DAVID) [19]. The species and background *Canis lupus familiaris* was selected for the functional annotations after uploading the genes. When submitting the gene list to the DAVID tool for functional annotation, we select an EASE score of 0.1 as the default choice and count threshold 3. The enrichment *p*-value in the functional annotation chart was determined based on the EASE score, and the *p*-value threshold (*p*-value of ≤0.05) for considerably significant enriched GO/KEGG terms was set [38,39].

## 3. Results

### 3.1. Phenotypic Data Analysis

We performed a GWAS with different quantitative traits such as height, length, LHR and body-weight in Jindo dog. We investigated 757 Jindo dogs with an average raw height of 48.51 cm, ranging from 33.5 to 59 cm and an average length of 51.42 cm ranging from 37 cm to 62 cm. The average LHR is 105.82, ranging from 86.4 to 130.7. The average raw weight in this individual dataset is 16.67 kg, ranging from 6.6 kg to 33.3 kg.

**Figure 1:**
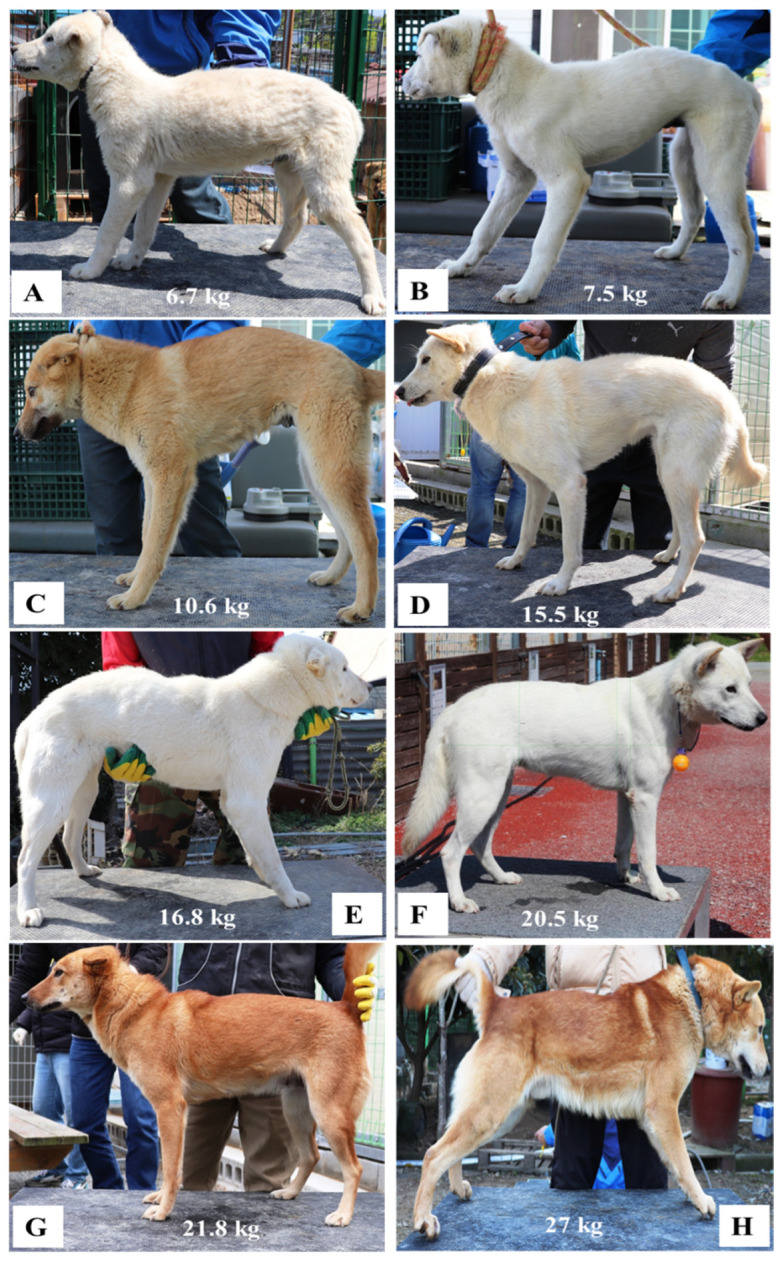
The phenotypic variation of Jindo dog. (**A**). Male Jindo dog with height 35.5 cm, length 38.5 cm, LHR 114.1, (**B**). Female Jindo dog with height 35 cm, length 40 cm, LHR 114.3, (**C**). Female Jindo dog with height 42 cm, length 43 cm, LHR 102.4, (**D**). Female Jindo dog with height 47 cm, length 48 cm, LHR 102.1, (**E**). Female Jindo dog with height 50 cm, length 54 cm, LHR 108, (**F**). Female Jindo dog with height 51.5 cm, length 54.4 cm, LHR 105.6, (**G**). Female Jindo dog with height 49 cm, length 52 cm, LHR 106.1, (**H**). Male Jindo dog with height 52.0 cm, length 55.5 cm, LHR 106.7.

The size variance was noticed to be high because of different ages of dogs were included in our current study for example a medium dog (younger than 16 months), adult dog (up to 8 years) and senior dogs (8 years or older). The standard error of the mean value was 0.13, 0.13, 0.18 and 0.16 for height, length, LHR and body weight, respectively. The distribution of dogs was done according to body-weight quartiles such as Q1 (the lowest 25% of registered weight)—8.4, Q2 and Q3—25% below (12.4) and above the median (20.8), Q4—dogs with the highest 25% of registered weight values (24.6). A total of 9 (Q1), 46 (Q2), 603 (Q3) and 99 (Q4) dogs were in each body-weight quartile. The ratios of genotypic and phenotypic variance explained by all the SNPs were found to be 0.33, 0.08, 0.08 and 0.28 for height, length, LHR and body weight, respectively. Figure 1 shows an example of phenotype measurement of male and female Jindo dogs. A descriptive statistical summary of the phenotypes included the minimum, maximum and mean, as well as the standard deviations and standard error are given in Table 1 and density plot for the traits is presented in Figure S1.

### 3.2. Genome-Wide Association Study

**Figure 2:**
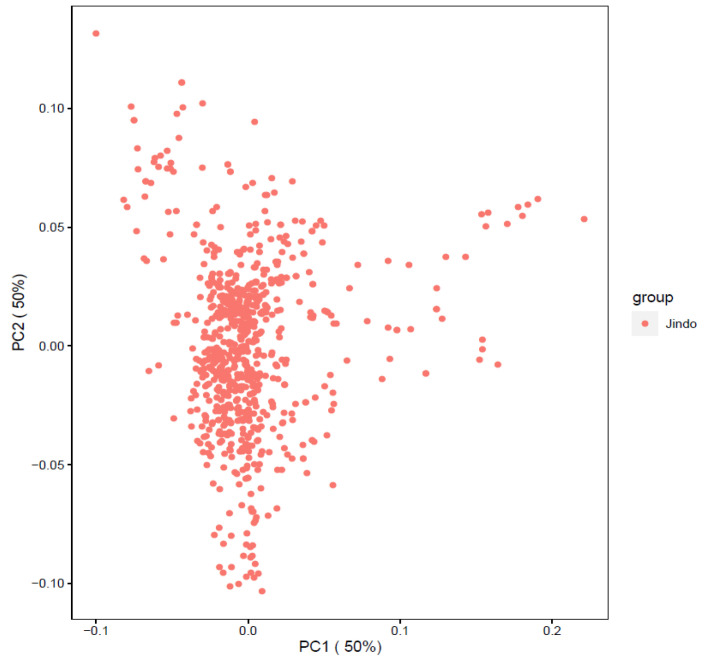
Population structure for all Jindo dog.

**Figure 3:**
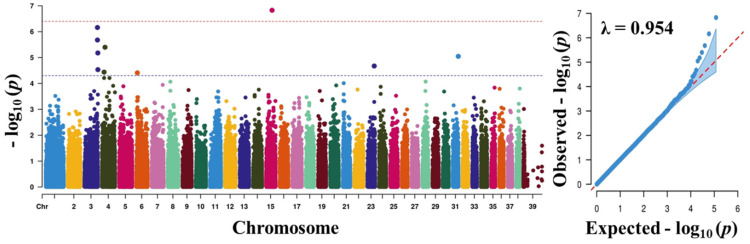
Manhattan plots of mapped single nucleotide polymorphism (SNP) markers associating with height. Red line designates the Bonferroni-corrected significance threshold level of *p* < 4 × 10^−^^7^ and blue line shows genome wide significant threshold level of *p* < 5 × 10^−^^5^. The quantile-quantile (Q-Q) plot of the GWAS is shown in the right side. GWAS, genome-wide association study.

**Figure 4:**
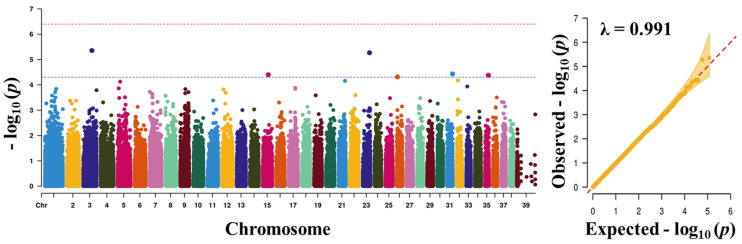
Manhattan plots of mapped single nucleotide polymorphism (SNP) markers associating with length. Red line designates the Bonferroni-corrected significance threshold level of *p* < 4× 10^−^^7^ and blue line shows genome wide significant threshold level of *p* < 5 × 10^−^^5^. The quantile-quantile (Q-Q) plot of the GWAS is shown in the right side. GWAS, genome-wide association study.

**Figure 5:**
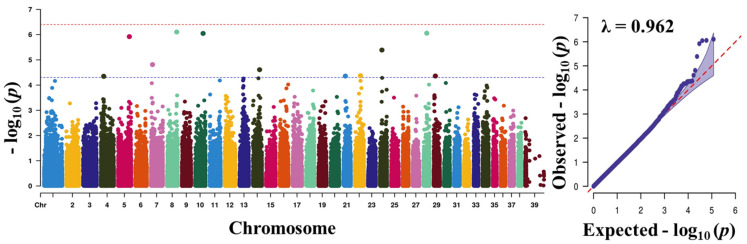
Manhattan plots of mapped single nucleotide polymorphism (SNP) markers associating with LHR. Red line designates the Bonferroni-corrected significance threshold level of *p* < 4 × 10^−^^7^ and blue line shows genome wide significant threshold level of *p* < 5 × 10^−^^5^. The quantile-quantile (Q-Q) plot of the GWAS is shown in the right side. GWAS, genome-wide association study. LHR, length to height ratio.

**Figure 6:**
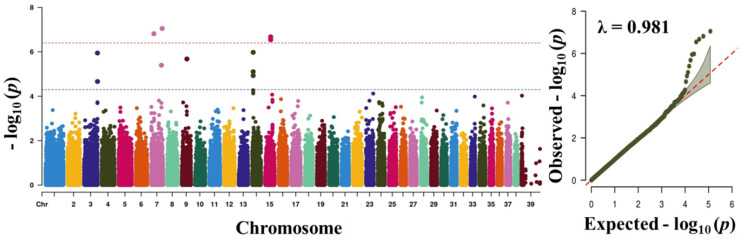
Manhattan plots of mapped single nucleotide polymorphism (SNP) markers associated with body weight. Red line designates the Bonferroni-corrected significance threshold level of *p* < 4 × 10^−^^7^ and blue line shows genome wide significant threshold level of *p* < 5 × 10^−^^5^. The quantile-quantile (Q-Q) plot of the GWAS is shown in the right side. GWAS, genome-wide association study.

Before carrying out the GWAS analysis, we analysed the patterns of population structure through principal component analysis (PCA) and the population found to form a good clustering pattern (Figure 2). We performed GWAS with a total of 757 dogs and 118,879 SNPs that passed after QC to discover significant loci associated with traits. The total genotyping rate was 0.988285. The Manhattan plot represents our mixed linear model-based GWAS result (Figure 3, Figure 4, Figure 5 and Figure 6). The association results revealed that a total of 40 SNPs in the genome were associated with all the four traits of height, length, LHR and body-weight at a suggestive significance level (*p* < 5 × 10^−^^5^). Among these SNPs, 10, 6, 13 and 11 SNPs were significant at the suggestive significance level (*p* < 5 × 10^−^^5^) for the trait of height, length, LHR and body-weight, respectively. A strong signal was identified only for height and body-weight trait, where one SNP (TIGRP2P201033_rs9187576) on Chr15 and 4 SNPs on Chr7 and Chr15 passed the Bonferroni-corrected significance threshold (*p* < 4 × 10^−7^). Most of these SNPs were located in the intronic regions and intergenic regions. No SNPs crossed Bonferroni-corrected genome-wide significance threshold (*p* < 4 × 10^−7^) for the traits of length and LHR. The top five significant (*p* < 5 × 10^−^^5^) associated SNPs and their annotated genes are presented in Table 2. For the height trait, top five SNPs (TIGRP2P201033_rs9187576, BICF2G630358507, BICF2G630358640, BICF2S23724992 and BICF2S2377250) were annotated to four genes (*HHIP*, *LCORL*, *NCAPG* and *P4HA1*), respectively. For length trait, the SNPs (BICF2P772349, BICF2P580549, BICF2S2366810, BICF2P517149 and BICF2G630778876) were annotated to four different genes such as *FGFR3*, *PLCH1*, *IGF1* and *SCAND3*, respectively. For LHR, the SNPs (TIGRP2P119932_rs8658799, BICF2G630268912, BICF2G630490347, BICF2P491431 and BICF2G630507820) were successfully annotated to seven genes (*BEGAIN*-*DLK1*, *TDRD1*, *EFEMP1*, *ZFHX3*-*PSMD7* and *SLC23A2*) genes, respectively. For body-weight, the SNPs (BICF2P279062, BICF2P979272, BICF2S23655947, BICF2S2336786 and BICF2P110929) were successfully annotated to seven genes (*PTPN2*, *RASAL2*, *PARPBP*-*IGF1*, IGF1, ENSCAFG00000001744-*SNORD19*) genes, respectively. The clear deviation between observed and expected *p*-values on the Q-Q plot for all the traits signifies good correlation results in the present study (Figure 3, Figure 4, Figure 5 and Figure 6).

### 3.3. Genotype-Phenotype Correlation

**Figure 7:**
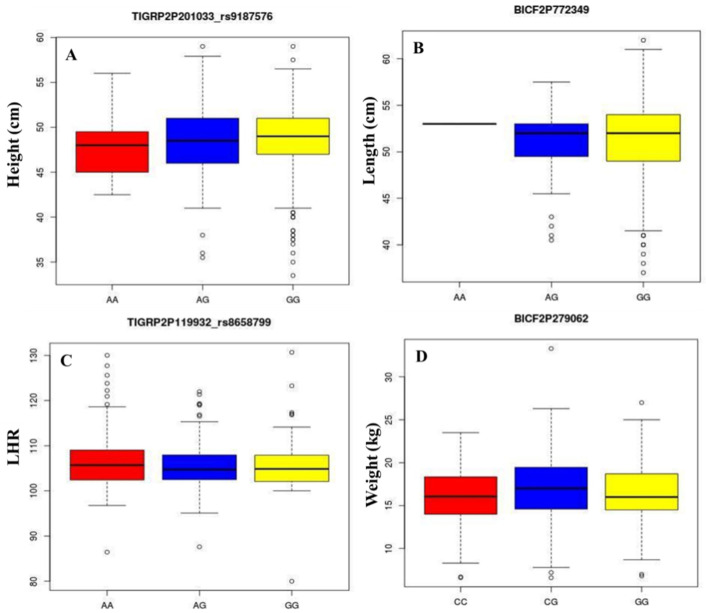
Genotype-phenotype correlation. The box plots show the distribution of phenotypes among the different genotypes of top variants associated to height (**A**), length (**B**), length to height ratio (**C**) and body-weight (**D**).

We acquired height, length, LHR and body-weight measurements on genotyped Jindo dogs. We then compared the traits (height, length, LHR and body-weight) distributions between the different genotype classes at the topmost variants, TIGRP2P201033_rs9187576, BICF2P772349, TIGRP2P119932_rs8658799 and BICF2P279062, respectively. The box plots show the distribution of phenotypes among the different genotypes (Figure 7). The solid lines in the box plot are the medians of phenotype’s per-genotype group (AA, AG, GG, CC, CG and CG) of all four variants, as described in Table 3. In addition, we calculated the mean height, length, LHR and body weight per genotype (Table 3). Although the differences between all three genotype classes were not significant, it showed that the A-allele for SNP TIGRP2P201033_rs9187576 was correlated with a mean reduction of the height trait in Jindo. For SNP BICF2P772349 and TIGRP2P119932_rs8658799, the A-allele showed to be correlated with increased length and LHR in Jindo dog, respectively. The trait-increasing allele A for SNP TIGRP2P119932_rs8658799 and G for SNP TIGRP2P201033_rs9187576 was found to be a major allele for both the SNPs. This A allele was detected as a minor allele in the case of BICF2P772349 SNP of the length trait. However, the AA genotype for SNP BICF2P772349 was found to be present only in a small number of total animals.

### 3.4. Gene-Set Enrichment and Pathway Analysis

Functional enrichment analysis was carried out to identify the classes of genes that are over-represented in a large group of genes and may have a connection with the studied phenotypes. Many genes are in GO term and KEGG pathway categories. From the GWAS result, 1222, 1132, 1102 and 1039 SNPs (nominal *p* < 0.01) were used to annotate 842, 817, 752 and 718 genes correlated with height, length, LHR and weight trait (Supplementary Table S1). Subsequently, the genes were uploaded to the bioinformatics tool, DAVID for finding the enriched GO terms and KEGG pathways. The analysis revealed that a total of 72 GO terms and 37 KEGG pathways for height, 69 GO terms and 19 KEGG pathways for length, 53 GO terms and 18 KEGG pathways for LHR, 50 GO terms and 25 KEGG pathways for weight traits were significantly (*p* < 0.05) enriched. Out of the total enriched GO terms and KEGG pathways, the top five significantly enriched GO terms and pathways are presented in Table 4.

**Figure 8:**
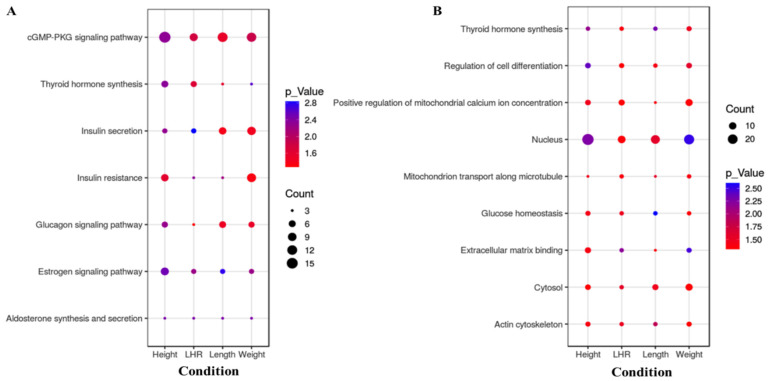
The significantly (*p* < 0.05) enriched 7 KEGG pathways (**A**) and 9 GO terms (**B**) shared among traits.

Moreover, we have highlighted here the significant enrichment pathways and GO terms that were overlapping among the traits. Among the total enriched GO terms and KEGG pathways, significantly (*p* < 0.05) enriched 9 GO terms and 7 pathways were found to be shared among traits (Figure 8). The enriched GO terms are GO:0005634−nucleus, GO:0047497−mitochondrion transport along the microtubule, GO:0051569−Thyroid hormone synthesis GO:0045595−regulation of cell differentiation, GO:0051561−positive regulation of mitochondrial calcium ion concentration, GO:0042593−glucose homeostasis, GO:0050840−extracellular matrix binding, GO:0015629−actin cytoskeleton, GO:0005829−cytosol. The KEGG pathways are cfa04911−Insulin secretion, cfa04915−Estrogen signalling pathway, cfa04925−Aldosterone synthesis and secretion, cfa04922−Glucagon signalling pathway, cfa04931−Insulin resistance, cfa04918−Thyroid hormone synthesis, cfa04022−cGMP-PKG signalling pathway.

## 4. Discussion

The present study reveals previously unreported information of genetic contribution to the important morphological phenotypes such as height, length, LHR and body weight of the Korean native breed, Jindo dog. Growth-related trait such as height, length and body weight are reported to have a higher heritability percentage [40]. Therefore, a higher heritable trait always needs to be precisely selected. Moreover, it has been shown in a survey study on the breeds’ height, bodyweight and related to data on 36 behavioural traits of companion dogs (*n* = 8301) of various common breeds (*n* = 49) collected internationally using the Canine Behavioural Assessment and Research Questionnaire (C-BARQ) that particular canine morphotypes tend to be reliably allied with particular behavioural profiles [41]. Here, marker-assisted selection can also be used to predict an animal’s phenotypic potential using molecular genetics [42]. Therefore, in this study we carried out GWAS supplemented with gene-set enrichment analysis to identify important functional candidate genes and pathways that influence the morphological phenotypes such as height, length, LHR and body-weight traits in Jindo dog and understand their regulatory roles.

We undertook an association study of 173,662 variants in 757 individual dogs and identified a total of 40 SNPs associated with height, length, LHR and body-weight traits at the genome-wide level (*p* < 5 × 10^−5^). A mixed-linear model has been used in this study to control the population stratification effect as it is the most effective method which simulates population structure, kinship and family structure [13,43]. Our association result revealed a highly significant association for the two traits such as height and weight. Here, we presented the top five significant SNPs (*p* < 5 × 10^−5^) associated with each trait (Table 1). The top leading significant variants are TIGRP2P201033_rs9187576 (height), BICF2P772349 (length), TIGRP2P119932_rs8658799 (LHR), BICF2P279062 (weight) which are located in the intronic region of *HHIP*, the upstream region of *FGFR3*, intergenic region of *BEGAIN*-*DLK1* and intronic region of *PTPN2* genes, respectively.

For the height trait, the *HHIP* gene (hedgehog interacting protein) involved in the development process has been previously reported to be associated with height and idiopathic short stature in children of the Korean population [44]. This gene has been detected for influencing bone and cartilage development, including skeletal development signalling pathway in humans [45,46]. Furthermore, *HHIP* is reported to play a role in lung branching morphogenesis during embryonic development [47]. Besides, our study enabled the identification of other noteworthy height-associated candidate genes including *LCORL* (ligand-dependent nuclear receptor corepressor like), *NCAPG* (non-smc condensin I complex subunit G) and *P4HA1* (prolyl 4-hydroxylase subunit alpha 1). The *LCORL* and *NCAPG* gene located on Chr3 was reported to be correlated with height by contributing to spermatogenesis and cell cycle [23,26]. These genes have constantly been recognised to influence human height in several large meta-analyses [48]. This *LCORL/NCAPG* locus has been previously identified for association with height and body-weight in horses and cattle [23,26]. The *LCORL* locus, in particular, has previously been found to be associated with both height and body weight in dogs, and it has been estimated that this gene accounts for 15% of the bodyweight variation in the dog population [14]. Furthermore, the *LCORL* gene has been reported to be involved in different conformation trait-like head, frame and neck development in horse as well as involved in trunk and hip development in human [49,50]. The *P4HA1* gene located on Chr 4, which correlates with Jindo dog’s height, is involved in collagen synthesis [23,29]. The *P4HA1* gene has not been reported in the dog before and the function of which in height regulation is also still unknown.

Bodyweight has been used as a surrogate for body size, which is the most striking trait for investigating genetic effects on quantitative traits. In human and even domestic animals, body size has been intensively studied [14,15,17,24,25,29,31,32,33]. Here, GWAS has helped us to identify candidate genes for body-weight in the Jindo breed. The protein tyrosine phosphatase non-receptor type 2 (*PTPN2*) gene which is located on Chr7, was stated to be involved in the regulation of development of diabetic periodontitis disease [51]. This result was in agreement with our previous GWAS study conducted on companion dog [20]. Another body-weight associated gene is *RASAL2* (RAS Protein Activator Like 2) gene located on Chr7 which has been found to be closely associated with adipogenesis process [52]. In fact, the *RASAL2* gene has been previously reported to be associated with increased body mass index in humans [53]. Furthermore, we found that *PARPBP* and *IGFI* genes are strongly associated with body-weight in Jindo dog. The only gene, *IGF1* was close to two SNPs (BICF2S23655947 and BICF2S2336786) located in the 41,134,849–41,239,301 bp region of Chr15. The insulin like growth factor-1 (*IGF1*) gene is a well-known candidate gene associated with size variation, which has been reported in every dog body size GWAS as well as those of many other mammals and, of course, humans [14,17,24,25,29,31,32,33].

*IGF1* binds to a type 1 IGF receptor, a signal transducer for a tyrosine kinase [32]. This communication stimulates the growth of cells and causes cellular differentiation for the survival of organisms [32]. The Poly [ADP-ribose] polymerase 1 binding protein-encoded gene, *PARPBP* has been reported before to be enriched in gene ontology terms, DNA repair and genome stability but the involvement in body-weight regulation is not yet confirmed [54]. The small nuclear RNA, C/D Box 19 (*SNORD19*) not reported previously to be associated with body weight, was detected on Chr14 in multiple regions. The role of this gene in body-weight regulation is still not identified.

Unlike height and body weight, length and LHR have not been well studied and less is known about its underlying genetics. Our results report new as well as previously reported genes associated with length or LHR. We observed 5 and 13 significantly correlated genetic variants for length and LHR, respectively. Among the 5 loci (*FGFR3*, *PLCH1*, *IGF1*, *SCAND3* and ENSCAFG00000008038), the *IGF1* gene on Chr15 was found to be common with body-size trait and had previously been reported in several studies [17,24,25,29,31,32,33]. Previously, a genome-wide fine-mapping study within the Portuguese water dog population found marked evidence for a single *IGF1* SNP haplotype which is a major contributor to small body size in all small dogs [32]. The *IGF1* gene may substantially affect dog length by regulating the development and functioning of joint, and controlling the body size [32,55]. The *FGFR3* gene encoding fibroblast growth factor receptor 3 located on Chr3 was the topmost associated candidate with length trait. Studies showed that fibroblast growth factor receptor is important for skeleton development [56]. Mutations in this gene cause human skeleton dysplasia, including developmental delay and achondroplasia [56,57]. Earlier, a selective sweep mapping study on detection of the genetic interval containing putative genes causing foreshortened limbs in Dachshunds dog by Pollinger et al., found a large sweep region in the vicinity of the *FGRF3* gene, proposing that the causative mutation in dogs is in a gene or regulatory region closely associated to *FGRF3* [58]. This condition can lead to short stature, macrocephaly, particularly in the proximal lower and upper limbs [56]. Among the 13 LHR associated genome-wide significant genes, the *EFEMP1* gene (EGF containing fibulin extracellular matrix protein 1) was showed to be associated with human height [59]. We identified another significant gene, *DLK1* on Chr8 which is one of the most strongly associated loci with LHR. The *DLK1* (delta-like non-canonical notch ligand 1) encodes a growth factor containing transmembrane protein which works as a controller of cell growth [60]. Furthermore, this gene is largely known for its contribution to adipogenesis and organism development [60].

Several of the genes that were observed to be associated with substantially linked SNPs in our study were enriched in GO terms, e.g., thyroid hormone synthesis, extracellular matrix binding, actin cytoskeleton (*p* < 0.05). Our result exposed the enrichment of gene-sets which worked together in a network to accomplish specific molecular processes [20]. Among the 9 GO terms, the nucleus was the enriched cellular component with the highest number of genes (Supplementary Table S2). It was previously found to be associated with weight trait in companion dogs [20]. The actin cytoskeleton is an essential cellular component for muscle development as it transforms myotubes into myofibers [13,61]. Muscle development is a complex process and it mainly depends on the hypertrophy and proliferation of muscle fibres [61]. The actin and actin-binding protein with another component of the cytoskeleton including the microtubule works together in a network to help essential cellular processes like axonal growth and cell migration [61]. The extracellular matrix has been associated with body-weight regulation as it helps cells in proliferation [62]. Thyroid hormone synthesis is closely correlated with growth and development [63]. It was noticed that the overlap in GO terms is largely housekeeping pathways, except for thyroid hormone synthesis. The genes enriched in thyroid hormone synthesis were *BDNF* (brain-derived neurotrophic factor), *CBLN1* (cerebellin 1 precursor), *SFRP1* (secreted frizzled-related protein 1), *IGF1* (insulin-like growth factor-1), *NTRK2* (neurotrophic receptor tyrosine kinase 2), *ACACA* (acetyl-coa carboxylase alpha), *FGFR3* (fibroblast growth factor receptor 3). Polymorphisms in two of these genes (*IGF1* and *FGFR3*) were found to be associated with body size and leg length in dogs, respectively [25,29,58]. Boule et al. described that the glucose homeostasis has potential role in weight regulation and it can predicts the weight gain [64].

The KEGG pathway analysis always has been used to access the group of genes associated with a particular trait. This method can identify enrichments by pooling data from multiple genetic SNPs, particularly through individual genetic SNP that do not meet a specific significance threshold [65]. Our KEGG pathway analysis has successfully detected several significantly enriched pathways. Some shared pathways between traits were noticed. It has been proposed that the regulation of correlated traits is more likely controlled by similar or related pathways [66]. Here, we identified seven overlapping pathways enriched to all traits (height, length, LHR and body-weight). Interestingly, six of those pathways were hormone-related pathways, such as the insulin secretion pathway, the oestrogen signalling pathway, the aldosterone synthesis and secretion, the glucagon signalling pathway, the insulin resistance pathway and the thyroid hormone synthesis pathway. The hormone is a key regulatory system for growth and development. Insulin and thyroid hormones are the most vital endogenous regulators of growth and skeletal development after growth hormone. The significance of the thyroid hormone for skeletal development and maintenance has been well documented in several studies [63]. The previous study showed that thyroid hormones control the metabolism of body proteins along with other hormones like growth hormone, insulin-like growth factor-1 (IGF-1), insulin and glucocorticoids, and thus regulate the growth and development process [63]. Insulin is a peptide hormone that has several effects on the metabolism of fats, proteins and carbohydrates [36]. Growth hormone receptor (GHR) sensitivity and the level of insulin-like growth factor 1 (IGF-1) can also be affected by insulin, consequently influencing the level of growth hormone [67]. The insulin resistance pathway has been previously reported to be associated with height and body-weight trait in humans [68]. The glucagon signalling pathway is a pathway that strongly affects the body-weight trait by increasing blood glucose level in the blood through glycogenolysis and gluconeogenesis [69]. In addition, we have identified another signalling pathway shared between traits that is the cGMP-PKG signalling pathway. The cGMP-PKG signalling pathway reported to be associated with contraction of vascular smooth muscle cells. It has previously been found in our previous study to be correlated with body-weight trait in dog [20].

## 5. Conclusions

In this study, we have discovered several new unreported as well as previously identified markers, genes and pathways that might influence the variation in height, length, LHR and body-weight of Jindo dog using GWAS with pathway-based approaches. The significantly associated markers harbouring *HHIP*, *LCORL*, *NCAPG*, *IGFI*, *FGFR3*, *DLK1*, *EFEMP1*, *PTPN2*, *IGFI* and *RASAL2* genes are probably the most attractive candidate genes as their functions are strongly related to height, length and body-weight phenotypes. Interestingly, the identified overlapping enriched pathways were found to be mainly hormone-related pathways (insulin secretion pathway, the oestrogen signalling pathway, the aldosterone synthesis and secretion, the glucagon signalling pathway, insulin resistance pathway and the thyroid hormone synthesis pathway) which participate in growth and development process. Integrating gene expression data along with our association data used in this study may expose the true genetic mechanisms underlying height, length and body-weight variation in Jindo dog.

## Figures and Tables

**Table 1:** Descriptive summary of phenotype data.

Traits		Phenotypic Data					Variance		
		492 Female, 265 Male							
	Mean	Median	Max	Min	SD	SE	h^2^	Vp	V(G)
Height (cm)	48.61	48.5	59	33.5	3.69	0.13	0.33	13.26	4.31
Length (cm)	51.42	51.3	62	37	3.62	0.13	0.08	106.68	8.81
LHR	105.82	105.6	130.7	86.4	5.23	0.18	0.08	361.40	32.14
Weight (Kg)	16.67	16.4	33.3	6.6	3.41	0.16	0.28	11.34	3.19

SD—standard deviation, SE—standard error, Vp—phenotypic variance, V(G)—genotypic variance, h^2^—heritability, LHR—length to height ratio.

**Table 2:** Top 5 SNPs associated with height, length, LHR and body weight in Jindo dog.

Trait	SNP ID	Chr	Position	*p*-Value	MAF	Gene	Type
Height	TIGRP2P201033_rs9187576	15	43493060	1.49 × 10^−7^	0.11	*HHIP*	Intron
	BICF2G630358507	3	91180492	6.91 × 10^−7^	0.47	*LCORL*	Intron
	BICF2G630358640	3	91314456	2.1 × 10^−6^	0.33	*NCAPG*	Intron
	BICF2S23724992	4	23625990	4 × 10^−6^	0.10	*P4HA1*	Intron
	BICF2S2377250	3	93890772	6.65 × 10^−6^	0.11	-	-
Length	BICF2P772349	3	62323843	4.42 × 10^−6^	0.29	*FGFR3*	Upstream_gene
	BICF2P580549	23	49471629	5.42 × 10^−6^	0.27	*PLCH1*	Intron
	BICF2S2366810	31	40675954	3.71 × 10^−5^	0.25	*-*	-
	BICF2P517149	15	41228320	3.98 × 10^−5^	0.24	*IGF1*	Intron
	BICF2G630778876	35	25711203	4.18 × 10^−5^	0.17	*SCAND3*	Intron
LHR	TIGRP2P119932_rs8658799	8	68874072	7.88 × 10^−7^	0.36	*BEGAIN-DLK1*	Intron
	BICF2G630268912	28	25034623	8.78 × 10^−7^	0.28	*TDRD1*	Intron
	BICF2G630490347	10	56634077	8.98 × 10^−7^	0.39	*EFEMP1*	Intron
	BICF2P491431	5	79708771	1.20 × 10^−6^	0.11	*ZFHX3-PSMD7*	Intergenic
	BICF2G630507820	24	16702908	4.09 × 10^−6^	0.07	*SLC23A2*	Intron
Weight	BICF2P279062	7	78202038	8.91 × 10^−8^	0.03	*PTPN2*	Intron
	BICF2P979272	7	21586876	1.54 × 10^−7^	0.37	*RASAL2*	Intron
	BICF2S23655947	15	41134849	2.12 × 10^−7^	0.05	*PARPBP-IGF1*	Intergenic
	BICF2S2336786	15	41239301	2.86 × 10^−7^	0.07	*IGF1*	Intron
	BICF2P110929	14	10389714	1.06 × 10^−6^	0.03	ENSCAFG00000001744-*SNORD19*	Intergenic

MAF—minor allele frequency, LHR—length to height ration.

**Table 3:** Effect of top SNP genotypes on the height, length, LHR and weight trait in Jindo dog.

Traits	SNP Name	Genotype	Counts	Mean	Median	SD
Height (cm)	TIGRP2P201033_rs9187576	AA	16	48.1	48.1	3.96
		AG	144	48.37	48.50	3.82
		GG	597	48.70	49	3.60
Length (cm)	BICF2P772349	AA	2	53.26	53.00	-
		AG	126	51.08	52.00	3.49
		GG	629	51.44	52.00	3.64
LHR	TIGRP2P119932_rs8658799	AA	306	105.93	104.90	4.98
		AG	349	105.69	104.86	4.32
		GG	102	105.67	104.85	5.86
Weight (kg)	BICF2P279062	CC	95	16.14	16.05	3.36
		CG	335	16.96	17	3.72
		GG	327	16.53	16	3.08

LHR—length to height ration.

**Table 4:** Top five Gene Ontology and KEGG pathways significantly enriched using genes associated with height, length, LHR and body weight.

Trait	Category	Term_ID	Term	Count	%	*p*-Value	Genes
Height	GOTERM_CC_DIRECT	GO:1902711	GABA-A receptor complex	6	0.0046	6 × 10^−4^	*GABRA2, GABRB2, GABRA6, GABRA5, GABRA4, GABRG1*
	GOTERM_MF_DIRECT	GO:0004890	GABA-A receptor activity	6	0.0046	7 × 10^−4^	*GABRA2, GABRB2, GABRA6, GABRA5, GABRA4, GABRG1*
	GOTERM_BP_DIRECT	GO:0006816	Calcium ion transport	8	0.0061	8 × 10^−4^	*PPP3CA, RYR2, CAMK2D, PLN, PRKCB, CCL5, CAV1, NMUR2*
	GOTERM_BP_DIRECT	GO:0030336	Negative regulation of cell migration	11	0.0084	9 × 10^−4^	*RNF20, KANK1, DACH1, STK24, DAG1, ADARB1, LDLRAD4, TBX5, SULF1, RECK, ROBO1*
	GOTERM_BP_DIRECT	GO:0045665	Negative regulation of neuron differentiation	9	0.0069	9 × 10^−4^	*DDX6, ZHX2, MEIS1, ID4, OLIG2, CNTN4, PBX1, GLI3, CDK5RAP2*
	KEGG_PATHWAY	cfa04024	Camp signalling pathway	23	0.0176	6 × 10^−6^	*GRIA1, RYR2, GABBR2, CAMK2D, CREBBP, SUCNR1, PPP1R12A, BDNF, HHIP, ADCY2, ADRB1, ATP2B1, GLI3, GNAI1, MAPK10, TIAM1, GRIN3A, PLN, FXYD2, CREB3L2, DRD1, CREB5, GRIA4*
	KEGG_PATHWAY	cfa04723	Retrograde endocannabinoid signalling	16	0.0122	1 × 10^−5^	*GABRA2, GRIA1, GABRB2, GABRA6, GABRA5, PRKCB, GABRA4, ADCY2, GNAI1, GRM1, GABRG1, MAPK10, RIMS1, GRM5, PLCB1, GRIA4*
	KEGG_PATHWAY	cfa04261	Adrenergic signalling in cardiomyocytes	17	0.0130	8 × 10^−5^	*RYR2, CAMK2D, PPP2R5A, ADCY2, ADRB1, ATP2B1, ADRA1B, GNAI1, PLN, PPP2R2B, PPP2R5E, KCNQ1, FXYD2, CREB3L2, PLCB1, CACNG3, CREB5*
	KEGG_PATHWAY	cfa05033	Nicotine addiction	9	0.0069	1 × 10^−4^	*GABRA2, GRIA1, GABRB2, GRIN3A, GABRA6, GABRA5, GABRA4, GRIA4, GABRG1*
	KEGG_PATHWAY	cfa04540	Gap junction	13	0.0010	2 × 10^−4^	*GUCY1A3, GUCY1B3, GUCY1A2, PRKCB, LPAR1, ADCY2, ADRB1, GNAI1, GRM1, TUBB6, GRM5, DRD1, PLCB1*
Length	GOTERM_CC_DIRECT	GO:0030425	Dendrite	21	2.7814	8 × 10^−7^	*GABRA2, EPHA4, SYT4, GABRA5, KCNB1, DENND1A, MAGI2, KLHL1, PDYN, GRM1, BRINP2, HTR7, RELN, PLCB4, BRINP1, GRM7, DNER, NCS1, MAPT, BRINP3, TP63*
	GOTERM_MF_DIRECT	GO:0005096	Gtpase activator activity	21	2.7814	8 × 10^−5^	*RGS18, RABGAP1, DAB2IP, ARHGAP18, RASAL2, ARFGAP3, ARHGAP15, ARHGAP24, TIAM2, TBC1D1, TBCK, TBC1D2, SIPA1L1, TBC1D21, DLC1, TBC1D13, RAPGEF2, PLCB1, DOCK1, GARNL3, RGS7*
	GOTERM_CC_DIRECT	GO:0043025	Neuronal cell body	15	1.9867	7 × 10^−4^	*GABRA2, DENND1A, SEZ6, KLHL1, GRIK2, PDYN, BRINP2, UCHL1, GRIN3A, BRINP1, DNER, RAPGEF2, APOB, ASIC2, BRINP3*
	GOTERM_BP_DIRECT	GO:0032332	Positive regulation of chondrocyte differentiation	5	0.6622	2 × 10^−3^	*PKDCC, SOX9, SOX6, GLI3, SOX5*
	GOTERM_BP_DIRECT	GO:0042593	Glucose homeostasis	3	1.6756	2 × 10^−3^	*TFAP2B, RFX6, MCU*
	KEGG_PATHWAY	cfa04922	Glucagon signalling pathway	6	1.6275	3 × 10^−2^	*PLCH1, ATP6V1G3, ACACA, CREB3L2, SLC2A2, CREB5*
	KEGG_PATHWAY	cfa04915	Oestrogen signalling pathway	4	2.385	2 × 10^−3^	*CREB3L2, ADCY8, GRM1, CREB5*
	KEGG_PATHWAY	cfa04024	cAMP signalling pathway	20	2.6490	1 × 10^−4^	*ABCC4, BDNF, CACNA1D, ADRB1, ADCY8, GRIN2B, GLI3, GNAI1, PPP1CC, TIAM1, GRIN3A, PLN, FXYD2, ORAI1, PLCE1, FFAR2, SOX9, CNGA4, GRIA4, RAPGEF4*
	KEGG_PATHWAY	cfa04918	Thyroid hormone synthesis	3	2.6375	3 × 10^−2^	*CREB5, PLCH1, SCAND3*
	KEGG_PATHWAY	cfa04080	Neuroactive ligand-receptor interaction	17	2.2516	4 × 10^−2^	*GABRA2, GABRB2, GRID1, GABRA5, ADRB1, TACR1, NMBR, GRIK2, PRL, GRIN2B, GRM1, GABRG1, GRIN3A, HTR7, GRM4, GRM7, GRIA4*
LHR	GOTERM_BP_DIRECT	GO:0048706	Embryonic skeletal system development	6	0.8535	3 × 10^−3^	*COL1A1, PCSK5, TAPT1, PBX1, ACVR2A, SULF2*
	GOTERM_BP_DIRECT	GO:0010595	Positive regulation of endothelial cell migration	6	0.8535	6 × 10^−3^	*BMPR2, ROCK2, PROX1, ETS1, BCAR1, VEGFA*
	GOTERM_MF_DIRECT	GO:0050840	Extracellular matrix binding	3	1.7065	6 × 10^−3^	*SPP1, SPARCL1*
	GOTERM_BP_DIRECT	GO:0048484	Enteric nervous system development	4	0.5689	8 × 10^−3^	*EDNRB, KIF26A, PHOX2B, PHACTR4*
	GOTERM_BP_DIRECT	GO:1901166	Neural crest cell migration involved in autonomic nervous system development	3	0.4268	9 × 10^−3^	*NRP1, NRP2, PHOX2B*
	KEGG_PATHWAY	cfa04911	Insulin secretion	4	0.8534	1 × 10^−3^	*CREB3L2, SLC2A2, ADCY8, CREB5*
	KEGG_PATHWAY	cfa04080	Neuroactive ligand-receptor interaction	20	2.8449	2 × 10^−3^	*NPFFR2, GRIA2, GABRA1, CHRNB4, CHRNA7, LPAR1, GRIK1, TACR1, NMBR, HTR4, GABRG3, ADRA2A, HCRTR2, GRM1, CRHR2, GHR, EDNRB, HRH2, APLNR, PRLHR*
	KEGG_PATHWAY	cfa04931	Insulin resistance	3	1.9914	5 × 10^−3^	*CREB5, SLC2A2, CREB3L2,*
	KEGG_PATHWAY	cfa04915	Oestrogen signalling pathway	4	1.5647	6 × 10^−3^	*CREB3L2, ADCY8, GRM1, CREB5*
	KEGG_PATHWAY	cfa05202	Transcriptional misregulation in cancer	13	1.8492	7 × 10^−3^	*CD86, PDGFA, ETV1, KLF3, FLI1, RELA, PBX1, ETV5, IGF1R, MEIS1, WT1, MYC, ERG*
Weight	GOTERM_CC_DIRECT	GO:0030425	Dendrite	16	2.3952	8 × 10^−5^	*GABRA2, KCNB1, DENND1A, HTR1D, KLHL1, NRG1, PDYN, GRM1, BRINP2, HTR7, PLCB4, BRINP1, DPYSL5, DPYSL2, NPTN, MAPT*
	GOTERM_MF_DIRECT	GO:0005096	Gtpase activator activity	18	2.6946	4 × 10^−4^	*RASAL2, SIPA1L3, RASGRP3, STXBP5L, SGSM1, TBC1D1, TBC1D2, ARHGAP31, SIPA1L1, TBC1D21, DLC1, TBC1D13, TBC1D12, PLCB1, ECT2, DOCK1, RGS6, RGS7*
	GOTERM_MF_DIRECT	GO:0017137	Rab gtpase binding	9	1.3473	6 × 10^−4^	*SGSM1, TBC1D1, TBC1D2, TBC1D21, TBC1D13, TBC1D12, MYRIP, RAB11FIP2, STXBP5L*
	GOTERM_BP_DIRECT	GO:0045666	Positive regulation of neuron differentiation	8	1.1976	2 × 10^−3^	*BRINP2, BMP2, TGIF2, BRINP1, GDF6, ECT2, PHOX2B, BEND6*
	GOTERM_BP_DIRECT	GO:0050852	T-cell receptor signalling pathway	7	1.0479	2 × 10^−3^	*CACNB4, PTPRC, CD28, FYN, GATA3, SHB, MALT1*
	KEGG_PATHWAY	cfa04921	Oxytocin signalling pathway	16	2.3952	2 × 10^−4^	*GUCY1A3, GUCY1B3, GUCY1A2, RYR2, KCNJ6, CAMK1D, CACNA2D1, ADCY2, PRKCA, PRKAB1, RCAN1, PLCB4, CACNB4, PLCB1, MYL9, CAMK1G*
	KEGG_PATHWAY	cfa05100	Bacterial invasion of epithelial cells	10	1.4970	2 × 10^−3^	*CDC42, FN1, ELMO1, CTNNA3, CTNNA2, WASL, PIK3CB, WASF1, DOCK1, PTK2*
	KEGG_PATHWAY	cfa04713	Circadian entrainment	11	1.6467	2 × 10^−3^	*GUCY1A3, GUCY1B3, PER1, GUCY1A2, RYR2, KCNJ6, GRIN2A, PLCB4, ADCY2, PRKCA, PLCB1*
	KEGG_PATHWAY	cfa04918	Thyroid hormone synthesis	3	1.1976	2 × 10^−3^	*CREB3L2, ADCY8, CREB5*
	KEGG_PATHWAY	cfa04520	Adherens junction	9	1.3473	4 × 10^−3^	*CDC42, PTPN1, PTPRM, CTNNA3, FYN, CTNNA2, WASL, WASF1, WASF3*

LHR—Length to height ration.

## Data Availability

The data presented in this study are available on request from the corresponding author.

## Supplementary Materials

The following are available online at https://www.mdpi.com/article/10.3390/ani11113136/s1. Figure S1: The density plot for the height, length, LHR and body-weight traits. Table S1: List of all SNPs (*p* < 0.01) associated with height, length, LHR and body-weight that were used for gene-set and pathway analysis. Table S2: List of all Gene Ontology and KEGG pathways significantly (*p* < 0.05) enriched using genes associated with height, length, LHR and body-weight.
