# Supplementary figures and images for: Insight into the Candidate Genes and Enriched Pathways Associated with Height, Length, Length to Height Ratio and Body-Weight of Korean Indigenous Breed, Jindo Dog Using Gene Set Enrichment-Based GWAS Analysis
Source: Animals (Basel). 2021 Nov 2;11(11):3136. doi: 10.3390/ani11113136 (PMC8614278; doi:10.3390/ani11113136)

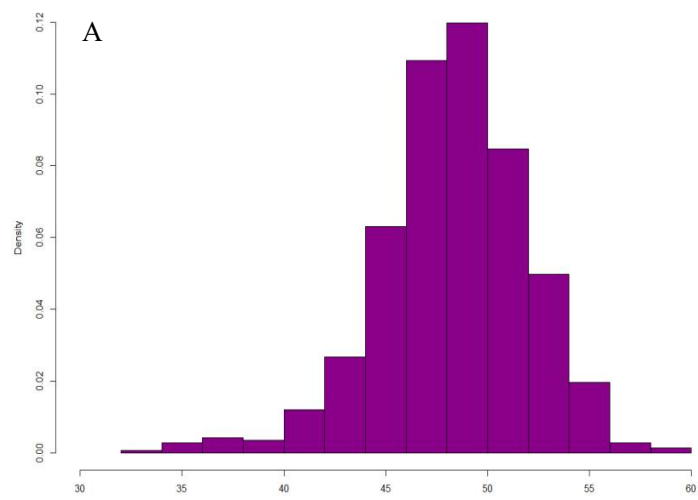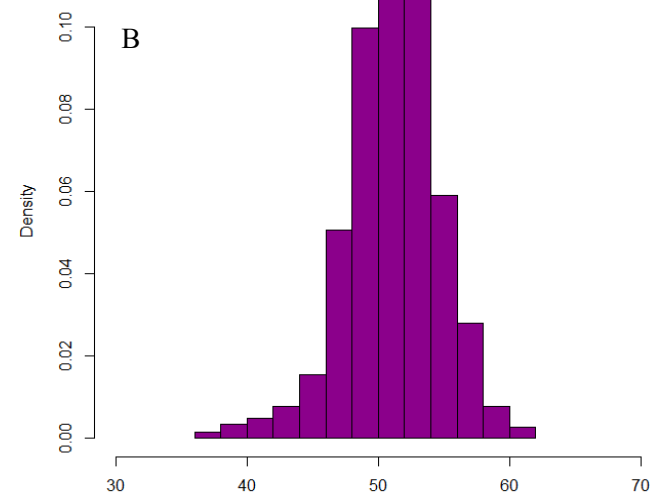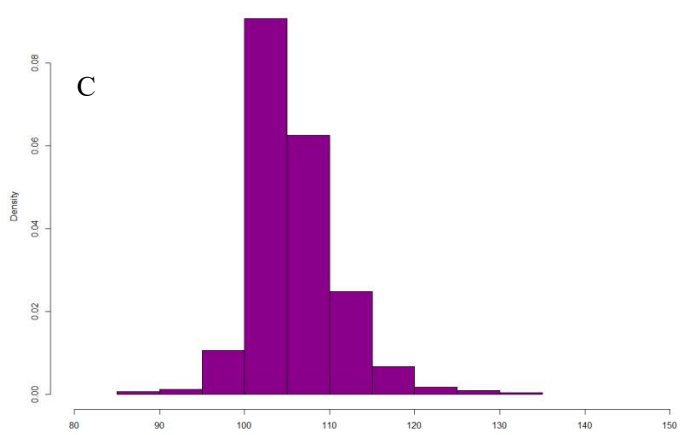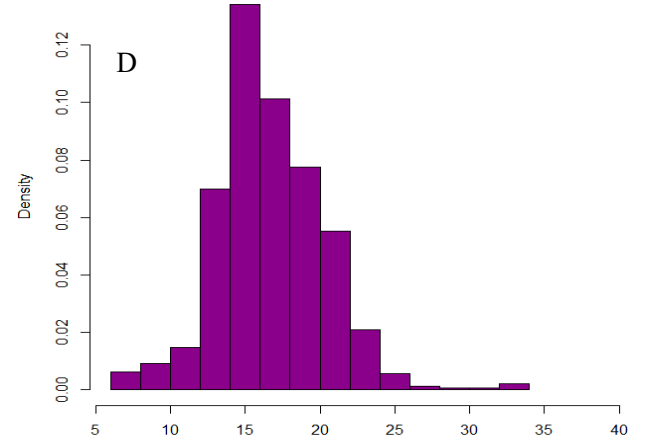

**Figure S1.** The density plot for the height (A), length (B), LHR (C), and body-weight (D) traits.

Supplement: Supplementary file 1 [file animals-11-03136-s001.zip › animals-1393291-supplementary/Supplementary Figure S1.pdf]
